# Systemic Sclerosis Immunoglobulin Induces Growth and a Pro-Fibrotic State in Vascular Smooth Muscle Cells through the Epidermal Growth Factor Receptor

**DOI:** 10.1371/journal.pone.0100035

**Published:** 2014-06-13

**Authors:** Monique R. Arts, Murray Baron, Nidaa Chokr, Marvin J. Fritzler, the Canadian Scleroderma Research Group (CSRG), Marc J. Servant

**Affiliations:** 1 Université de Montréal, Faculté de Pharmacie, Montreal, Quebec, Canada; 2 McGill University, Jewish General Hospital, Montreal, Quebec, Canada; 3 Department of Medicine, University of Calgary, Calgary, Alberta, Canada; Institute of Immunology, Rikshospitalet, Norway

## Abstract

**Objective:**

It has been suggested that autoantibodies in systemic sclerosis (SSc) may induce the differentiation of cultured fibroblasts into myofibroblasts through platelet-derived growth factor receptor (PDGFR) activation. The present study aims to characterize the effects of SSc IgG on vascular smooth muscle cells (VSMCs) and to determine if stimulatory autoantibodies directed to the PDGFR can be detected, and whether they induce a profibrotic response in primary cultured VSMCs.

**Methods:**

Cultured VSMCs were exposed to IgG fractions purified from SSc-patient or control sera. VSMC responses were then analyzed for ERK1/2 and Akt phosphorylation, PDGFR immunoprecipitation, cellular proliferation, protein synthesis, and pro-fibrotic changes in mRNA expression.

**Results:**

Stimulatory activity in IgG fractions was more prevalent and intense in the SSc samples. SSc IgG immunoprecipitated the PDGFR with greater avidity than control IgG. Interestingly, activation of downstream signaling events (e.g. Akt, ERK1/2) was independent of PDGFR activity, but required functional EGFR. We also detected increased protein synthesis in response to SSc IgG (p<0.001) and pro-fibrotic changes in gene expression (*Tgfb1* +200%; *Tgfb2* −23%; *p*<0.001)) in VSMCs treated with SSc IgG.

**Conclusion:**

When compared to control IgG, SSc IgG have a higher stimulation index in VSMCs. Although SSc IgG interact with the PDGFR, the observed remodeling signaling events occur through the EGFR in VSMC. Our data thus favour a model of transactivation of the EGFR by SSc-derived PDGFR autoantibodies and suggest the use of EGFR inhibitors in future target identification studies in the field of SSc.

## Introduction

Systemic sclerosis (SSc) is a chronic disorder of connective tissue characterized by autoimmunity, inflammation, fibrosis and vascular disease. Although the aetiology of this disease is poorly understood, possible contributory factors include agonistic autoantibodies that target receptors, which in turn may contribute to the SSc pathology. One such autoantibody has been reported to target and activate the platelet-derived growth factor receptor (PDGFR) on fibroblast cells, leading to downstream signaling activity and culminating in pro-fibrotic events [1]. Such autoantibodies were not detected in any controls but were in all SSc patients tested and also in graft-versus-host disease [2], two conditions characterized by fibrosis and autoimmunity. The B-cell depleting drug Rituximab, which may have beneficial effects on lung function and skin fibrosis in SSc patients [3], has been associated with reduced PDGFR phosphorylation in SSc skin [3], supporting the possibility of agonistic anti-PDGFR-autoantibodies. In contrast, others have reported that both healthy and SSc patients possess such autoantibodies, and/or that such autoantibodies do not lead to signaling activity [4]–[6]. PDGFR-activating autoantibodies have also been reported in systemic lupus erythematosus (SLE) patients [7].

Proliferative and obstructive vasculopathy is common in SSc. Most patients experience Raynaud's phenomenon, and many develop vascular ulcers of the extremities [8]. Pulmonary arterial hypertension (PAH) in SSc is a leading cause of mortality and affects 10–12% of patients [9]. PAH is characterized by increased pulmonary vascular resistance and features uncontrolled endothelial and vascular smooth muscle cell (VSMC) growth, vasoconstriction and extracellular matrix accumulation, thus obstructing the pulmonary arterial circulation [9], [10]. VSMCs are known to express PDGFRs and in these cells PDGF is a mitogen [11]. Moreover, PDGFR-β immunoreactivity was reported to be more prevalent and intense in the pulmonary vessels of a SSc-PAH group than in controls [12]. Thus, if PDGFRs on VSMCs are activated by anti-PDGFR autoantibodies present in the circulation of SSc patients, this could contribute to the ubiquitous vascular pathology of the disease. We therefore sought to determine if SSc sera contain antibodies that stimulate VSMCs and if so, whether these antibodies are directed to PDGF receptors.

It is also noteworthy that the expression levels of both the PDGFR and epidermal growth factor receptor (EGFR) are significantly increased in the vasculature of SSc-PAH patients compared to controls [12]. In a molecular context, a certain proportion of these receptors are expressed as heterodimers in VSMCs and interestingly, the activation of early signaling events by PDGF in these cells occurs through transactivation of the EGFR via a process that is independent of PDGFR kinase activity but dependent on EGFR kinase activity [13], [14]. Thus, we investigated whether the EGFR might play a role in VSMC stimulation by SSc IgG. It is also known that Angiotensin II acts as a growth factor and pro-inflammatory cytokine for cultured VSMCs through binding to the Angiotensin II Type I receptor (AT1R) [15], [16]; thus this G protein-coupled receptor is recognized as a major vascular remodeling effector [17]. Interestingly, transactivation of the PDGFR by the AT1R has been shown to be involved in VSMC signaling events [18]. Moreover, activating anti-AT1R autoantibodies have been reported in SSc [19]. We therefore also investigated the possibility that SSc IgG may stimulate VSMCs through the AT1R.

## Patients and Methods

### Ethics Statement

All serum samples were obtained from patients or control subjects who had provided written informed consent for the use of their biological samples. The study was approved by the ethics committee of the Université de Montréal (CERSS#919) and by the ethics board of the SMBD Jewish General Hospital, Montreal, Quebec, Canada. Our use of animal-derived cells was approved by the Animal Ethics Committee of the Université de Montréal (protocol #09-156) and conformed to the Guide for the Care and Use of Laboratory Animals.

### Patients and biological samples

Serum samples from 23 SSc patients were obtained from the biobank of the Canadian Scleroderma Research Group (CSRG), maintained at the University of Calgary. For this study we required that subjects have early (5 years or less since onset of first non-Raynaud's symptom) diffuse cutaneous SSc according to 1980 ACR preliminary classification criteria [20], and not be on any immunosuppressive or steroid therapy. Prospective data collected on each patient by their rheumatologists at the time of the patient's visit, as previously described [8], included: Modified Rodnan skin score [21], Medsger vascular disease severity [22], presence of any active vascular cutaneous ulcers, pulmonary hypertension (PH; defined as an estimated systolic pulmonary arterial pressure (PAP)≥45 mmHg on echocardiogram which correlates strongly with right heart catheter studies [23], [24]), and presence of anti-centromere, anti-topoisomerase, and anti-RNA Polymerase III antibodies. Anti-centromere antibodies were detected by indirect immunofluorescence staining of HEp-2 cell substrates (ImmunoConcepts Inc., Sacramento, CA), while anti-topoisomerase antibodies were measured by an addressable laser bead immunoassay using an INOVA ENA 9 QuantaPlex kit (INOVA Diagnostics, San Diego, CA) and a Luminex 100 illuminometer (Luminex Corp., Austin, TX), and antibodies to RNA Polymerase III were detected by ELISA (INOVA) [25].

Controls from CSRG-participating clinics consisted of five age- and sex-matched otherwise healthy individuals with osteoarthritis who were not on corticosteroids or immunosuppressives, seven normal healthy individuals, one SLE patient and one sample from a normal pooled blood bank.

### Immunoglobulin purification

IgG was purified from serum using Immobilized Protein A/G (Pierce, Rockford, IL) according to manufacturer recommendations, scaled down for use with Handee Mini Spin Columns (Pierce). All binding, wash and elution steps were performed by gravity-flow. The flow-through was reapplied three times, and the bound protein washed three times with 1 M NaCl and 8 times with binding buffer (Pierce). Eluted IgG was immediately neutralized with Tris-HCl (pH 8.5) and concentrated using Amicon 100 kDa centrifugation tubes (Millipore, Billerica, MA) according to manufacturer instructions, thereby excluding any growth factors or other molecules smaller than 100 kDa. To verify the efficiency of IgG purification and the quality of purified IgG samples, eluates were examined by 10% acrylamide SDS-PAGE followed by Coomassie blue staining. A bicinchoninic acid (BCA) assay (Pierce) was used to determine concentration of IgG samples. Samples were aliquoted and stored at −80°C until required for use in cell stimulation experiments.

### Reagents, antibodies and pharmacological inhibitors

Recombinant human PDGF-BB and EGF were obtained from Biosource (Camarillo, CA). Recombinant rat PDGF-BB and recombinant human TGF-β1 were purchased from R&D Systems (Minneapolis, MN). Angiotensin II was from Sigma (St. Louis, MO). The following pharmacological inhibitors were used in our study: PDGFR inhibitors AG1296 (Calbiochem, Gibbstown, NJ) and imatinib mesylate (Alexis Biochemicals, San Diego, CA); AT1R inhibitor irbesartan (a kind gift from Dr. Pierre Moreau, Université de Montréal); and EGFR inhibitor AG1478 (Biomol, Plymouth Meeting, PA).

Antibodies specific for Phospho-p44/42 MAPK (pERK1/2) (Thr202-Tyr204) and p44/42 MAP Kinase (ERK1/2), Phospho-Akt (Ser473), Akt, Phospho-EGF Receptor (Tyr845) and EGF Receptor (C74B9) were from Cell Signaling Technology (Beverly, MA). Anti-Mouse CD140a (PDGFR a) and CD140b (PDGFR b) Functional Grade Purified neutralizing antibodies were obtained from eBioscience (San Diego, CA). Anti-PDGFRα and β antibodies were from Upstate Biotechnology (Lake Placid, NY).

### Cell culture

Aortic VSMCs were isolated from Wistar rats by explant and maintained in high-glucose DMEM supplemented with 10% fetal bovine serum (FBS). MRC5 cells were obtained from ATCC, and cultured according to supplier recommendations. Cells at 75% confluence were rendered quiescent by incubation for 48 hours in serum-free high glucose DMEM and Ham's F-12 (1∶1) supplemented with 15 mM Hepes (pH 7.4), 0.1% low-endotoxin bovine serum albumin (Sigma), and 5 µg/mL transferrin (Sigma) for 48 h. Experiments were conducted on cells at passages 5–13. For experiments with pharmacological inhibitors, cells were pre-treated for 30 minutes with vehicle alone or with the indicated concentrations of inhibitors.

Cells were maintained in the absence of antibiotics and routinely tested for mycoplasma contamination using LookOut™ Mycoplasma PCR Detection Kit (Sigma) or MycoAlert™ Mycoplasma Detection Kit (Lonza, Rockland, ME).

### Immunoblot analyses

350,000 VSMCs were seeded in 6-well plates in DMEM/10%FBS. Quiescent cells were stimulated with 200 µg/mL purified IgG [1] for 5 minutes, at 37°C under 5% CO_2_. After 5 minutes, cells were gently washed two times with ice-cold PBS and whole cell extracts were prepared using Triton-X lysis buffer (50 mM Tris-HCl, pH 7.4, 150 mM NaCl, 50 mM NaF, 5 mM EDTA, 40 mM β-glycerophosphate, 1 mM sodium orthovanadate, 0.2 mM phenylmethylsulfonyl fluoride (PMSF), 1 µg/mL leupeptin, 1 µM pepstatin A, 2 µg/mL aprotinin, 1% Triton-X-100, 10% glycerol) for 30 min at 4°C. Lysed material was then centrifuged at 13,000× *g* for 10 min and the supernatant collected. Equal amounts of lysate proteins (20–50 µg) were loaded on 7.5 or 10% polyacrylamide gels and subjected to SDS-PAGE. In some Western blot experiments, cellular extracts were divided and used in parallel. Proteins were transferred to nitrocellulose membranes in 25 mM Tris, 192 mM glycine and 20% methanol using a Bio-Rad Transblot Cell transfer apparatus. Immunoblotting with each antibody was carried out according to manufacturer instructions.

Western blot bands were analyzed by densitometry using ImageQuant TL version 2002 (Amersham, NJ). Densities of p-ERK bands were normalized to corresponding total ERK bands. A stimulation index was determined for each sample using the equation (S-C)/(P-C)×100 where S, C, and P represent the normalized band intensities of a given sample, the negative control and the positive control, respectively [1]. In addition, we measured the phosphorylation of ERK2 by ELISA, using DuoSet IC: Human/Mouse/Rat Phospho-ERK2 (T185/Y187) ELISA kit (R&D Systems) according to manufacturer recommendations.

### Immunoprecipitation assays

Quiescent VSMCs were lysed on ice, using a RIPA buffer (50 mM Tris-HCl (pH 7.4 at 4°C), 150 mM NaCl, 5 mM EDTA, 50 mM NaF, 40 mM β-glycerophosphate, 1% Triton-X-100, 10% glycerol, 0.1% SDS, and 1% Na-deoxycholate, 1 mM sodium orthovanadate, 1 µg/mL pepstatin, 2 µg/mL aprotinin, 1 µg/mL leupeptin, 0.2 mM PMSF) for 30 minutes. 500 µg of whole cell extracts were incubated for 4 hours at 4°C with 2 µg of anti-PDGFR-β (Upstate), 200 µg SSc or control IgG immobilized on 50 µL protein-A-Sepharose beads (GE Healthcare). The immune complexes were washed four times with Triton X-100 lysis buffer and 2X Laemmli's sample buffer was added. The immunoprecipitated proteins were analysed by immunoblotting using commercial anti-PDGFR-β antibodies.

### RT-qPCR analysis

150,000 VSMCs were seeded in 6-well plates in DMEM/10%FBS. Quiescent cells were stimulated with 200 µg/mL purified IgG [1] for 2 hours (*col1a1* and *colIII*) or 72 hours (*Tgfb1*, *Tgfb2*, *Tgfb3*), at 37°C under 5% CO_2_. Cells were then gently washed with ice-cold PBS and flash-frozen in liquid nitrogen. Next, cell lysates were collected and total RNA isolated using RNeasy Mini Kit (QIAGEN) according to manufacturer directions, and spectrophotometric quantification of RNA samples was performed. RNA integrity was verified using a Bioanalyzer system at the Institute for Research in Immunology and Cancer (IRIC; Montreal). Total RNA (2 µg) was reverse-transcribed using the High Capacity cDNA Reverse Transcription kit with random primers (Applied Biosystems) as described by the manufacturer. Real-time PCRs were subsequently performed using the Fast SYBR Green Master Mix (Applied Biosystems) with the following primers: ***colIII***: FWD 5′-AGATGCTGGTGCTGAGAAG-3′; REV 5′-TGGAAAGAAGTCTGAGGAAGG-3′; ***Tgfb1***: FWD 5′-CCTGGAAAGGGCTCAACAC-3′; REV 5′-CAGTTCTTCTCTGTGGAGCTGA-3′; ***Tgfb2***: FWD 5′-AGTGGGCAGCTTTTGCTC-3′; REV 5′-GTAGAAAGTGGGCGGGAT G-3′; ***Tgfb3***: FWD 5′-AGTGGCTGTTGCGGAGAG-3′; REV 5′-GCTGAAAGGTATGACATGGACA-3′; ***Actb***: FWD 5′-CCCGCGAGTACAACCTTCT-3′; REV 5′-CGTCATCCATGGCGAACT-3′ (UPL probe #17). Quantitect primer assay (QIAGEN) was used for ***col1a1***. β-actin was used as endogenous control. Samples were initially denatured at 95°C for 3 min followed by 40 cycles of 5 sec at 95°C and 30 sec at 60°C. All reactions were run in triplicate and the average threshold cycle (Ct) values were used for quantification. The relative quantification of target genes was determined using the DDCT method [26]. Briefly, the Ct values of target genes were normalized to those of an endogenous control gene, β-actin (DCt = Ct_target_−Ct_CTRL_) and compared with a calibrator: DDCT = DCt_Sample_−DCt_Calibrator_. Relative expression (RQ) was calculated as RQ = 2^−DDCT^.

### [^3^H]-leucine and [^3^H]-thymidine incorporation assays

45,000 or 60,000 VSMCs were seeded per well in 24-well plates for [^3^H]-leucine and [^3^H]-thymidine incorporation assays, respectively. After 24 hours, cells were rendered quiescent for 48 hours, then stimulated in triplicate with positive controls or purified IgG samples (200 µg/mL). For cell proliferation assays, [^3^H]-thymidine (MP Biomedicals) was added to a final concentration of 0.5 µCi/mL, and for protein synthesis assays, [^3^H]-leucine (MP Biomedicals) was added to a final concentration of 0.3 µCi/mL. Cells were then incubated for 24 hours at 37°C, 5% CO_2_. After 24 hours, media was removed, and cells were washed and fixed overnight at 4°C with ice-cold 5% tri-chloro-acetic acid (Fisher). Plates were gently washed with water and once dry, the cells were solubilized in 0.1N NaOH and added to scintillation tubes containing EcoLite(+) scintillation cocktail (MP Biomedicals), and vortexed vigorously. Radioactivity was measured using a Tri-Carb 2100TR Liquid Scintillation Analyzer (Packard) as counts per minute (CPM) per well and expressed as fold change from basal.

### Statistical analyses

Statistical analyses were performed using GraphPad Prism version 5.0 for Mac (GraphPad Software, San Diego, CA). Comparison of two groups was carried out by two-tailed t-test, and comparison of more than two groups was carried out with one-way ANOVA and a Bonferroni post-test. Statistical significance was accepted at *P*≤0.05.

## Results

### Patients

We studied sera from 23 SSc patients with a disease duration of 5 years or less since first non-Raynaud's symptom and not on any steroid or immunosuppressive therapy. General characteristics of our patient cohort are summarized in Table 1 (details of individual subject characteristics are available in Table S1). 91% of patients were female. The mean age was 48.7 years. The mean modified Rodnan Skin Score was 16.5 and the mean Medsger vascular disease severity score was 1.9. 36% of SSc patients had active vascular ulcers and 4.8% had pulmonary hypertension on echocardiography. 19% of patients had anti-centromere antibodies, and 29% had anti-topoisomerase antibodies. A large proportion, 43%, of our patients had anti-RNA polymerase III antibodies, which is not surprising since this antibody is more common in diffuse cutaneous SSc [25], [27]. 8.7% of our patient group had received some immunosuppressive treatment less than a year prior to having their blood drawn. The 13 controls consisted of 5 normal subjects, 5 osteoarthritis patients, one SLE patient and one sample from a normal pooled blood bank. The mean age of controls was 43.6 (SD = 16.5) and 83% were females.

**Table 1:** Characteristics of our SSc cohort (N = 23).

	% or mean ±sd
Mean age, years	48.7±14.5
Female	91%
Disease duration, years	3.5±1.2
Mean Modified Rodnan Skin Score	16.5±11.2
Mean Medsger vascular disease severity	1.9±1.3
Subjects with active vascular ulcers	36%
Subjects with pulmonary hypertension	4.8%
Subjects with anti-centromere antibodies	19%
Subjects with anti-topoisomerase antibodies	29%
Subjects with anti-RNA polymerase III antibodies	43%
Subjects with recent use of immunosuppressive drugs	8.7%

### Purified IgG from SSc sera have a greater stimulation index in VSMC than normal IgG

**Figure 1:**
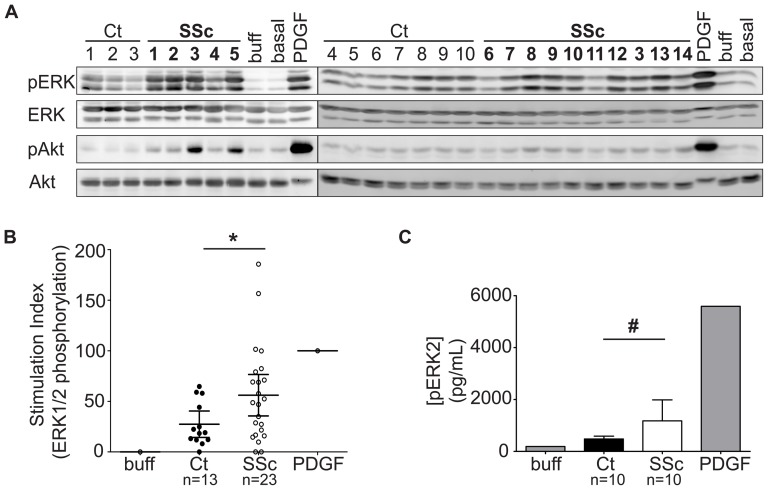
Effects of purified scleroderma (SSc) and control (Ct) IgG on signaling activity in vascular smooth muscle cells (VSMCs). **A**, Representative Western blots showing ERK1/2 and Akt phosphorylation in quiescent VSMCs treated for 5 minutes with 200 µg/mL purified IgG, the IgG buffer alone, or with 50 ng/mL PDGF. **B**, Densitometric quantification of all immunoblot bands, expressed as a stimulation index scaled to the positive control (PDGF; stimulation index of 100) and negative control (buffer; stimulation index of 0) on the same gel, from 13 control and 23 SSc samples. The stimulation index is calculated as (S-C)/(P-C)×100 where S, C, and P represent the normalized densitometric pERK1/2 band intensities of a given Sample, the negative Control and the Positive control, respectively. The mean stimulation with 95% confidence intervals is indicated. * *p* = 0.0474. **C**, ELISA analysis of ERK2 phosphorylation in VSMC stimulated with 200 µg/mL SSc IgG (n = 10) or control IgG (n = 10). Error bars represent 95% confidence interval. *p* = 0.0559. buff =  buffer; PDGF =  platelet-derived growth factor.

Quiescent VSMCs were exposed for 5 minutes to 200 µg/mL IgG purified from SSc and control sera, and ERK1/2 and Akt phosphorylation were analyzed as an indication of activation of early signaling pathways involved in vascular remodeling events [28], [29]. As previously conducted [1], a stimulation index was determined for each sample (Table S1) by densitometric analyses of Western blots (Fig. 1A). A comparison of the mean stimulation activity of SSc IgG with that of control IgG revealed a higher stimulation capacity in the SSc samples (*p* = 0.0474; Fig. 1B), and the highest levels of ERK1/2 and Akt phosphorylation were observed in cells treated with SSc IgG (Fig. 1B).

In addition to immunoblot analyses, we measured by ELISA the ability of a subset of IgG samples (SSc subjects 3 and 6–15) to induce ERK2 phosphorylation. Again, the increase in ERK2 phosphorylation in SSc-IgG-treated VSMCs was greater than in control-IgG-treated cells (Fig. 1C). Using sera from 2 SSc subjects, cells were exposed to 50, 100, 150 and 200 µg/mL IgG. The increase in ERK1/2 phosphorylation was dose-dependent (Fig. S1).

The purified IgG samples had the same effects on other cell lines. Although we studied the effects of SSc IgG mainly in VSMCs obtained from rat cells, the increased phosphorylations of Akt also occurred in cells of human origin. Primary diploid human fibroblasts (MRC5 cells) exposed to different SSc IgG fractions exhibited increased Akt phosphorylation, similar to that seen in rat VSMCs (Fig. S2).

### PDGFR-β interacts more avidly with SSc IgG than control IgG but does not mediate the increase in ERK1/2 and Akt signalling

**Figure 2:**
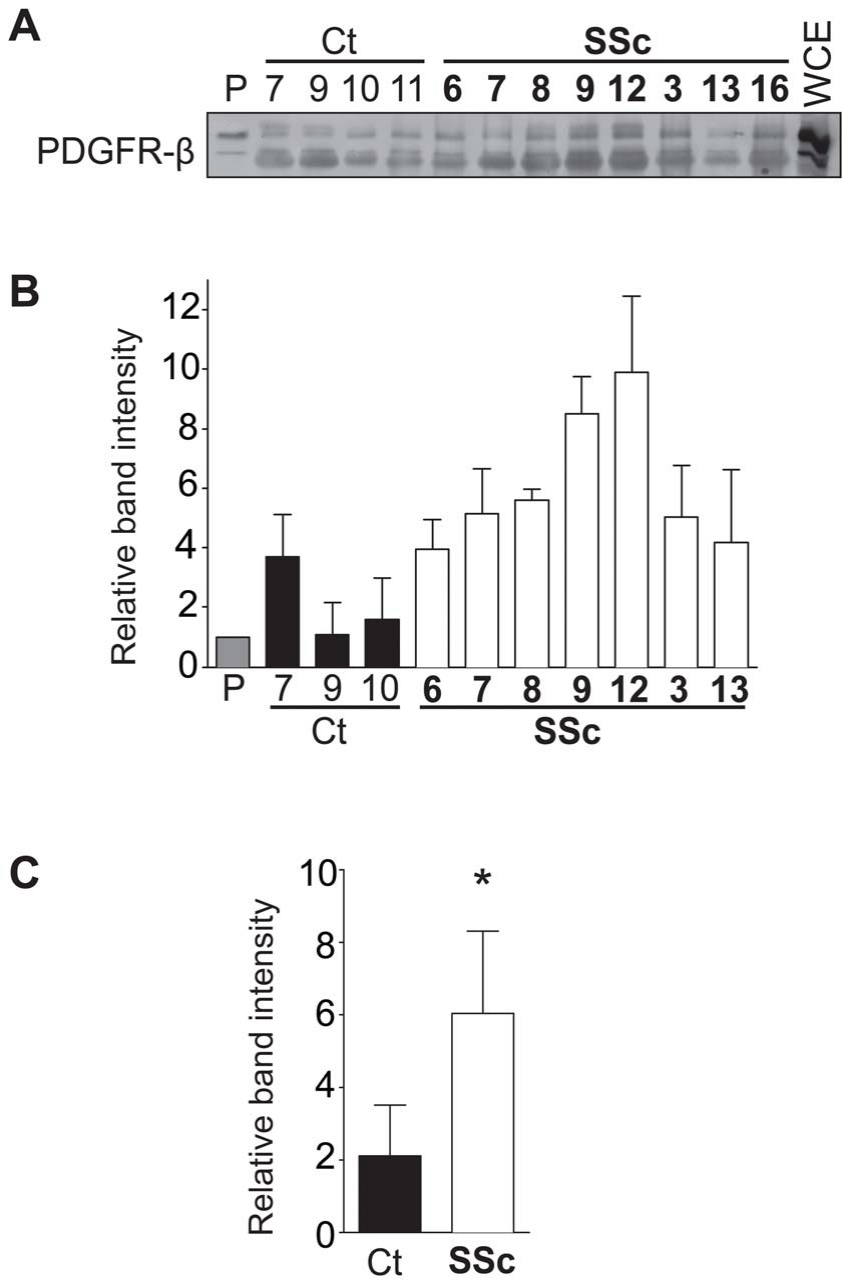
IgG from systemic sclerosis (SSc) patients bind more to PDGFR than control (Ct) IgG. **A**, Representative immunoblot of PDGFR-β immunoprecipitated with different control or SSc IgG samples, or with a commercially available PDGFR-β antibody (P). The whole cell extract (WCE) was loaded in the far right lane. **B**, Average densitometric analysis of immunoblots from two separate experiments. **C**, Comparison of mean band intensities for control and SSc samples. * *p* = 0.0358.

In order to address the ability of SSc IgG to recognize and specifically bind the PDGFR, we next performed immunoprecipitation studies using purified IgG from a subset of 8 SSc and 7 control subjects (Representative data shown in Fig. 2A, B). Although there was variation among samples, on average, the SSc-IgG immunoprecipitated significantly more PDGFR-β than control IgG (*p* = 0.0385; Fig. 2C). We did not observe any statistically significant correlation between the stimulation index of individual samples (Table S1) and the capacity to immunoprecipitate the PDGFR-β.

**Figure 3:**
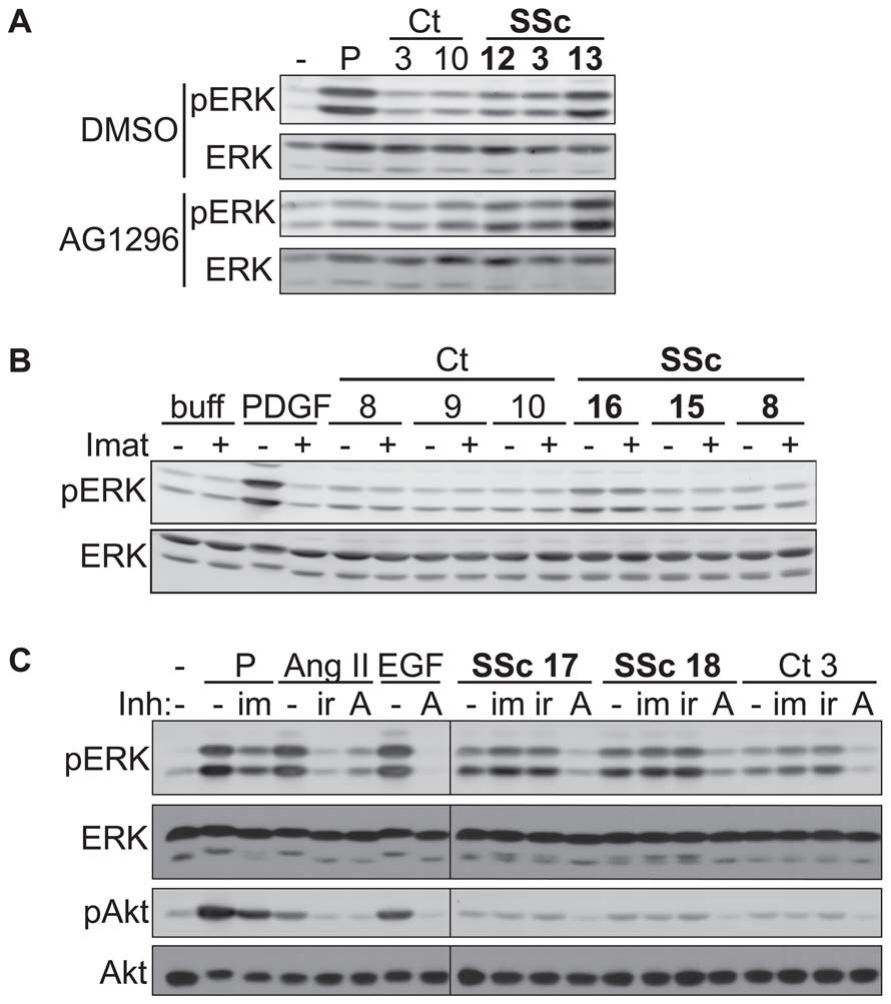
The phosphotranferase activity of EGFR plays a key role in SSc IgG-induced early signaling events in VSMCs. **A**, Quiescent vascular smooth muscle cells (VSMCs) were pre-treated for 30 minutes with vehicle (0.01% DMSO) or PDGFR-inhibitor AG1296 (5 µM) before stimulation with 50 ng/mL PDGF (P) or 200 µg/mL control (Ct) or systemic sclerosis (SSc) patient IgG. **B**, Quiescent VSMCs were pre-treated for 60 minutes with 10 µM imatinib mesylate (Imat; +) or with 0.01% DMSO (−), followed by 5-minute stimulation with 50 ng/mL PDGF or 200 µg/mL IgG from control or SSc patients. **C**, Quiescent VSMCs were pre-treated with inhibitors (Inh; 10 µM imatinib mesylate (im), 1 µM irbesartan (ir), 250 nM AG1478 (A)), or 0.01% DMSO (−) for 30 minutes prior to stimulation for 5 minutes with 50 ng/mL PDGF (P), 100 nM angiotensin II (AngII), 100 ng/mL epidermal growth factor (EGF), or 200 µg/mL SSc or control IgG. All results shown are representative of at least two experiments with similar results.

We next investigated whether the catalytic activity of the PDGFR was involved in SSc-IgG-induced early signaling events in VSMC. The quinoxaline AG1296 is a highly potent and selective inhibitor of the PDGFR-α and β isoforms and its family members c-kit and flt3 [30], [31]. Cells pre-treated with AG1296 had indeed a dramatic reduction in ERK1/2 phosphorylation upon stimulation with PDGF-BB. However, SSc-IgG-induced ERK1/2 and Akt phospho-signals were not affected by the use of this PDGFR kinase inhibitor (Fig. 3A, Fig. S3). To further substantiate these observations, cells were also pre-treated with imatinib mesylate, which, in addition to blocking the kinase activity of c-Abl also antagonizes the PDGFR and c-kit [32]. Once again, the observed SSc-IgG-induced activation of ERK1/2 and Akt was not affected by this drug although it diminished the response to PDGF-BB stimulation (Fig. 3B, C). Thus, despite the ability of the SSc-IgG fractions to recognize and stably interact with the PDGFR-β, our data suggest that they do not use the kinase activity of this receptor family to transmit downstream intracellular signals.

### Epidermal growth factor receptor (EGFR), but not the AT1R, plays a key role in the VSMC response to SSc IgG

Since anti-AT1R autoantibodies have been demonstrated in SSc [19] we tested the AT1R antagonist irbesartan in SSc-IgG stimulated cells. In our study, the AT1R antagonist irbesartan abolished Ang II signaling in VSMC but did not affect SSc IgG-induced ERK and Akt phosphorylation (Fig. 3C). The potent and selective EGFR kinase inhibitor AG1478 [33] was also tested. At very low concentration, this compound significantly reduced EGF-induced phosphorylation of ERK1/2 and Akt (Fig. 3C). As previously shown [34], AG1478 also diminished the ability of Ang II to induce ERK1/2 and Akt activation resulting from AT1R-EGFR transactivation [18]. More importantly, blocking the EGFR phospho-transferase activity severely affected SSc IgG-induced activation of these early signaling events, suggesting a role for the EGFR's catalytic activity in the cellular response to SSc IgG (Fig. 3C).

### SSc IgG causes growth and pro-fibrotic responses in VSMCs

**Figure 4:**
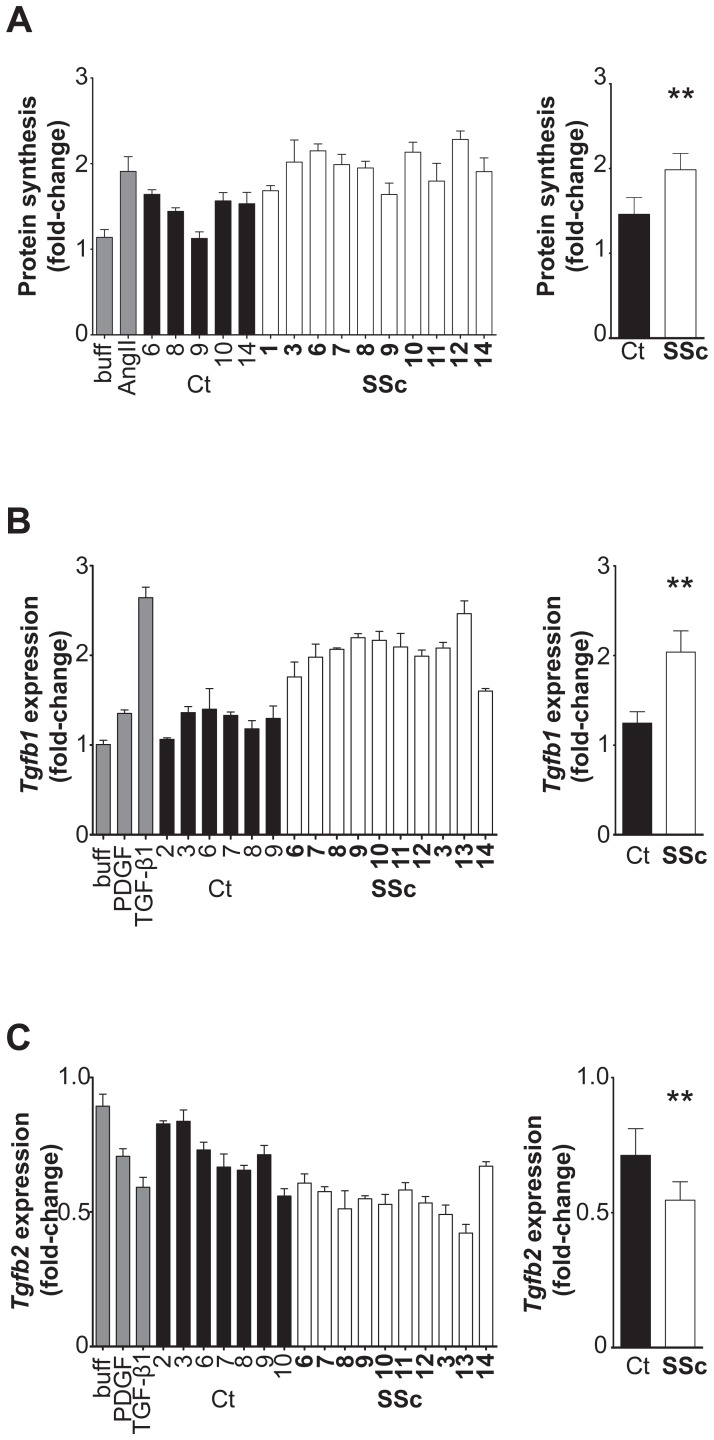
Systemic sclerosis (SSc) IgG causes growth and pro-fibrotic responses in vascular smooth muscle cells (VSMCs). **A**, Protein synthesis was measured by [^3^H]-leucine incorporation in quiescent VSMCs stimulated with IgG buffer, angiotensin II (AngII), or with 200 µg/mL SSc or control (Ct) IgG (left), and mean protein synthesis was compared for Ct-IgG and SSc-IgG treated cells (right). Protein synthesis is shown as a fold-change with respect to basal levels. ** *p* = 0.0008. **B**, Expression of transforming growth factor-β1 (TGF-β1) mRNA (*Tgfb1*) in VSMCs upon treatment with IgG buffer (buff), 50 ng/mL platelet-derived growth factor (PDGF), 10 ng/mL TGF-β1, or 200 µg/mL IgG for 2 hours (left). Results are normalized to *β-actin* expression and expressed as a fold-change with respect to untreated VSMCs. Mean TGF-β1 expression in Ct-IgG-treated VSMCs compared with that in SSc-IgG treated cells (right). ** *p*<0.0001. **C**. Expression of TGF-β2 mRNA (*Tgfb2*) in VSMCs upon treatment with IgG buffer, 50 ng/mL PDGF, 10 ng/mL TGF-β1, or IgG for 2 hours (left). Results are normalized to *β-actin* and expressed as a fold-change with respect to untreated VSMCs. Mean TGF-β2 expression in Ct-IgG-treated VSMCs compared with that in SSc-IgG treated cells (right). ** *p* = 0.0009. All results shown are representative of at least two experiments. Error bars represent standard deviation.

To address the potential of SSc IgG to cause pathophysiological changes in VSMCs that are linked to vascular remodeling events, we measured changes in cell growth and proliferation. These experiments were conducted with a subset of our SSc (n = 10) and control IgG samples (n = 5). Cell proliferation studies, as measured by incorporation of radiolabeled thymidine after incubation of VSMC with 200 µg/mL IgG, indicated that neither SSc nor control IgG caused any detectable increase in DNA synthesis (data not shown). Purified IgG from both controls and SSc patients did cause an increase in protein synthesis as measured by radiolabeled leucine incorporation, but protein synthesis was greater in VSMCs stimulated with SSc IgG (Fig. 4A). While on average, control IgG increased protein synthesis by 46% above basal levels, SSc IgG caused a mean increase of 93% (*p* = 0.0008).

Given the fibrotic features of SSc, we measured the expression of genes with roles in fibrosis, specifically *Col1a1*, *ColIII*, and *Tgfb1*, *2*, and *3*. Most importantly, we found that VSMCs responded to stimulation with purified SSc IgG by modulating the gene expression of TGF-β isoforms 1 and 2 (Fig. 4B, C). TGF-β is considered a master regulator of fibrotic processes [35]. Cells were stimulated with PDGF and TGF-β1, which modulates its own gene expression, as positive controls. Again, the IgG buffer solution alone had no detectable effect on the expression of any of the three protein isoforms. *Tgfb1* expression was induced significantly by all SSc IgG samples tested, and was induced to a lesser degree by some, but not all, control IgG samples (Fig. 4B). On average, SSc IgG caused a 2-fold induction of *Tgfb1* in VSMCs, which was significantly higher than control IgG (*p*<0.0001). In contrast, *Tgfb2* expression was decreased in cells treated with SSc IgG (*p* = 0.0009); Fig. 4C). *Tgfb3* gene expression was not affected by treatment with SSc IgG (data not shown). Also, SSc IgG did not affect expression of the collagen genes, *col1A1* or *colIII* in VSMCs after 72 h (data not shown), despite this being the time point at which PDGF and TGF-β caused the greatest modulations in *col1A1* or *colIII* gene expression in these cells (data not shown).

### Relationship to disease phenotype

Patients were grouped according to various disease manifestations (e.g. presence or absence of vascular ulcers, level of disease severity, mRSS, autoantibodies, pulmonary hypertension, etc.) and compared for differences in assay results (e.g. IgG stimulating activity, protein synthesis, etc), but no significant differences were found between groups (data not shown). Similarly, past use of immunosuppressive therapy did not have any effect on the cell-based assay results.

## Discussion

Our study demonstrated that SSc IgG induced growth and profibrotic responses in cultured VSMCs, which are known contributors to obstructive vasculopathy. We found that exposure of VSMCs to SSc IgG led to activation of protein kinases known to regulate vascular remodeling events. Activation of PDGFR and/or EGFR leads to initiation of numerous signaling cascades, including the ERK1/2 and Akt pathways [36], [37]. In VSMCs, ERK1/2 has been shown to be a crucial signaling molecule involved in various aspects of vascular remodelling, including the control of TGF-β gene expression, thus the fibrotic response, upon PDGF stimulation [28], and the protein kinase Akt regulates numerous cellular processes, including protein synthesis [38]–[40]. The increases in ERK1/2 and Akt phosphorylation that we observed were not inhibited by PDGFR inhibitors but were inhibited by an EGFR inhibitor, despite SSc IgG binding to PDGFR-β. Also, there was greater protein synthesis in VSMCs stimulated with SSc IgG, as well as simultaneous *Tgfb1* upregulation and *Tgfb2* downregulation, in response to VSMC exposure to SSc IgG. TGF-β1 is generally considered to have important pro-fibrotic roles in fibrosis, while the TGF-β2 isoform has been described as anti-fibrotic. Decreased production of TGF-β2 is associated with increased expression of a collagen mRNA variant in avian scleroderma, thus its downregulation would lead to a pro-fibrotic state [41].

A small number of studies have addressed the presence of functional activating autoantibodies in SSc, albeit with seemingly contradictory results [1], [4]–[6], [19]. Baroni *et al.* found unique and necessary presence of autoantibodies in SSc directed to the PDGFR on fibroblasts [1], while other studies found no difference in PDGFR-binding antibodies between control and SSc groups [5], [6] and/or no agonistic activity at all [4], [5]. In our cell-based assays, we found variability among the tested samples. Although all SSc and Ct samples immunoprecipitated the PDGFR-β to some degree, certain SSc IgG samples bound the receptor with much greater affinity than the controls. Similarly, the cellular responses to IgG stimulation were more pronounced in SSc IgG-treated cells.

**Figure 5:**
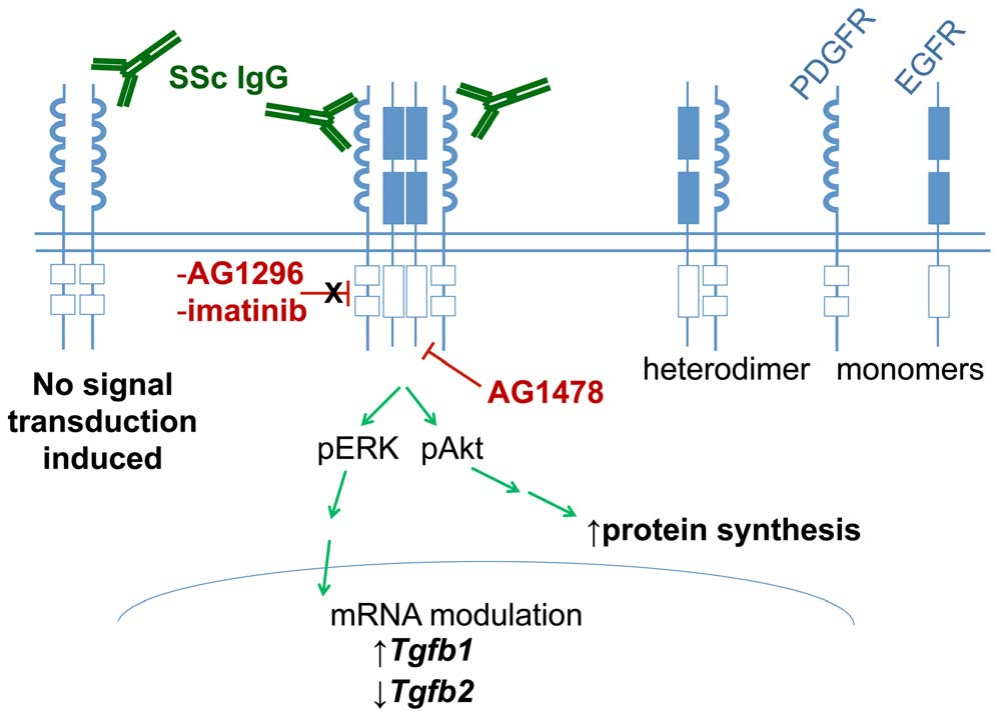
Platelet-derived growth factor receptor (PDGFR) and epidermal growth factor receptor (EGFR) are expressed as heterodimers in vascular smooth muscle cells (VSMCs) and interestingly, the activation of early signaling events by PDGF in these cells occurs through transactivation of the EGFR via a process that is independent of PDGFR kinase activity but dependent on EGFR kinase activity*. In this context, our data suggest that SSc IgG may bind to the PDGFR allowing transactivation of the EGFR leading to activation of early signaling events, increased protein synthesis, and differential regulation of TGFβ1 and TGFβ2 mRNA expression. The PDGFR inhibiting drugs, AG1296 and imatinib mesylate, do not disrupt SSc-IgG induced ERK1/2 and Akt phosphorylation. EGFR-blocking AG1478 inhibits SSc-IgG induced ERK1/2 and Akt phosphorylation. Image adapted from: *Saito Y, Haendeler J, Hojo Y, Yamamoto K, Berk BC. Receptor heterodimerization: essential mechanism for platelet-derived growth factor-induced epidermal growth factor receptor transactivation. Mol Cell Biol. 2001;21(19):6387-94.

The use of PDGFR kinase inhibitors, however, did not interrupt the signaling activity that we observed suggesting that, although there was PDGFR-binding, this was not the mechanism of action of stimulatory IgG on these cells. Also, it has been reported that SSc autoantibodies can have functional activity on AT1Rs in endothelial cells [19], but we were not able to detect autoantibodies that stimulated signaling through the AT1R in our VSMCs. We did, however, observe signaling that required the catalytic activity of the EGFR. The EGFR is known to have trans-activating activity [42], [43] and has also been reported to heterodimerize with other receptors, including the PDGFR [13]. Interestingly, PDGFR and EGFR are constitutively expressed as heterodimers in primary cultured VSMCs and the activation of early signaling events by PDGF in these cells occurs through the transactivation of the EGFR via a process that is independent of PDGFR kinase activity but dependent on EGFR kinase activity [13]. Heterodimerization of these two receptors has also been shown in a bladder cancer cell line transfected with the PDGFR-β gene [44]. PDGF has also been reported to trans-activate EGFR in skin fibroblasts and in cell-free membranes [45]–[47]. Indeed, we have also observed PDGFR-EGFR heterodimers in quiescent VSMCs in our lab as previously reported by Saito *et al.* [13] (Fig. S4). Although SSc-IgG-induced signaling was not blocked with PDGFR kinase inhibitors or an AT1R antagonist, it is conceivable that the EGFR signaling that we observed is one part of a multi-receptor signaling system that may include PDGFR (Fig. 5). In this context, we propose that SSc IgG may engage the PDGFR, a process leading to EGFR activation and subsequent induction of cellular signaling events in a manner independent of the enzymatic activity of the PDGFR, like previously proposed [13]. Our study supports these interesting findings and extends them by showing that this can also occur with activating autoantibodies. How the PDGFR affects the EGFR in the absence of its kinase activity is still unknown but could involve the tyrosine kinase c-Src [14]. In experimental models of SSc, the selective Src kinase inhibitor SU6656 reduced the development of dermal fibrosis [48]. Thus, targeting of Src kinases may be another promising approach in the treatment of SSc.

EGFR-binding autoantibodies have previously been reported in SSc patients, as well as in SLE patients and autoimmune mice [49], but those antibodies had neutralizing or inhibitory effects on A431 cells with the inhibition of DNA synthesis. Taken together with the finding that in VSMCs, a significant proportion of EGFRs and PDGFRs exist as heterodimers [13] and tests in our lab that show no significant difference in EGFR immunoprecipitation with SSc compared to control IgG (data not shown), we consider unlikely that anti-EGFR autoantibodies are responsible for our observations.

Interestingly, a recent study described SSc IgG that bind to permeabilised VSMCs and induce VSMC contraction [50]. Although the relationship between IgG binding to the VSMC and VSMC reactivity was not discussed, it is known that EGFR activation leads to Ca^2+^ release from intracellular stores [51], and subsequent vascular contraction [52].

The VSMC response to stimulation with purified IgG is consistent with SSc characteristics such as the changes in gene expression reflecting pro-fibrotic changes. TGF-β1 is a pro-fibrotic cytokine and its up-regulation is clearly linked with increased fibrosis [53]. TGF-β2 has been reported to have anti-fibrotic effects in the avian scleroderma model [41], its presence being linked to reduced expression of a pro-collagen mRNA variant. Thus our finding that TGF-β2 is down-regulated by SSc IgG is consistent with a pro-fibrotic state. Also, the increased protein synthesis we observed reflects cell growth and may be relevant in the context of PAH, for example.

We did not detect any relationship between the patient phenotype and stimulatory activity in terms of ERK1/2 or Akt phosphorylation, protein synthesis, gene expression changes, or receptor binding. However, our sample size was relatively small for this type of analysis and we do not feel that any firm conclusions should be drawn until much larger numbers of patients are studied. This would allow better evaluation of the clinical significance of these autoantibodies and of the relationship of these antibodies with the clinical manifestations of SSc.

The presence of increased numbers of autoantibodies define autoimmune diseases and recently autoantibodies have been investigated for functional pathogenic activity. The functional autoantibodies which we describe may have a role in initiating and/or perpetuating the vascular disease of SSc. Further studies are required to better understand the role of the EGFR and to determine if and how the PDGFR and EGFR might interact in response to autoantibody binding. Finally, the rather disappointing results from the use of the non-selective c-Abl, PDGFR, c-kit inhibitor imatinib in SSc clinical trials ([54]–[56] and reviewed in [57]) may in part be explained by the involvement of EGFR, as we observed in our study. Thus, it may be worthwhile to examine the effects of EGFR inhibitors on the course of the vascular disease of SSc.

## Supporting Information

Figure S1 SSc IgG causes increased ERK phosphorylation in quiescent vascular smooth muscle cells in a dose-dependent manner. Cells were exposed to 50, 100, 150 and in the case of SSc 5 200 µg/mL IgG.
(TIF)

Figure S2 Effects of purified scleroderma (SSc) and control (Ct) IgG on signaling activity in a primary human fibroblast cell line (MRC5), after 5 minutes of exposure to 200 µg/mL purified IgG, 50 ng/mL PDGF, or the IgG buffer.
(TIF)

Figure S3 The platelet-derived growth factor receptor (PDGFR) inhibitor AG1296 does not inhibit the phosphorylation of ERK or Akt in vascular smooth muscle cells (VSMCs) stimulated with IgG from systemic sclerosis (SSc) patients. The inhibitor was effective in completely reducing the signal in response to stimulation with PDGF (compare PDGF/DMSO (lane 13) vs PDGF/AG1296 (lane 11)). Quiescent VSMCs were pre-treated for 30 minutes with vehicle (0.01% DMSO) or PDGFR-inhibitor AG1296 (5 µM) before stimulation with 50 ng/mL PDGF (P) or 200 µg/mL control (Ct) or scleroderma (SSc) IgG.
(TIF)

Figure S4 Constitutive heterodimerization of epidermal growth factor receptor (EGFR) and platelet-derived growth factor receptor (PDGFR) in quiescent vascular smooth muscle cells (VSMCs). Quiescent VSMCs were treated with the cross-linker 1-Ethyl-3-[3-dimethylaminopropyl]carbodiimide hydrochloride (EDAC) for 20 or 30 minutes. Cell lysates were prepared as described (Mol Cell Biol. 2001;21(19):6387-94C) and proteins were immunoprecipitated with anti-EGFR antibodies and immunoblotted for PDGFR-β.
(PDF)

Table S1 Details of individual patient samples. ANA, type of anti-nuclear antibodies detected; PH, pulmonary hypertension; DSS, Medsger disease severity score (0 = normal, 1 = mild, 2 = moderate, 3 = severe, 4 = endstage); mRSS, modified Rodnan skin score; ^1^T, anti-topoisomerase I; ^2^R, anti-RNA Polymerase III; ^3^0, none detected; ^4^n.a., data not available; ^5^C, anti-centromere; ^6^recent  =  having been stopped less than one year prior to blood draw; ^7^“not recent”  =  having been used at any time more than a year prior to blood draw; CS, corticosteroids; HCQ, hydroxychloroquine; MTX, methotrexate; ^8^stimulation index is calculated as (S-C)/(P-C)×100 where S, C, and P represent the normalized densitometric pERK1/2 band intensities of a given Sample, the negative Control and the Positive control, respectively.
(PDF)
