# Supplementary figures and images for: Systemic Sclerosis Immunoglobulin Induces Growth and a Pro-Fibrotic State in Vascular Smooth Muscle Cells through the Epidermal Growth Factor Receptor
Source: PLoS One. 2014 Jun 13;9(6):e100035. doi: 10.1371/journal.pone.0100035 (PMC4057313; doi:10.1371/journal.pone.0100035)

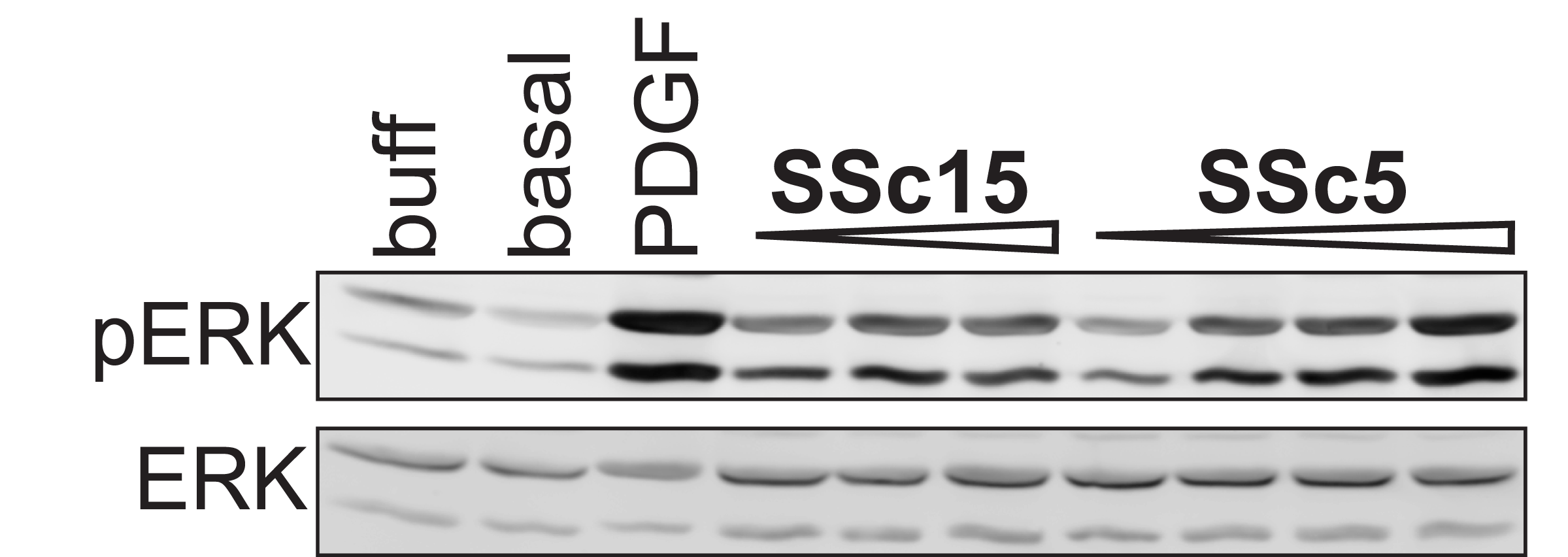

Supplement: Figure S1 — SSc IgG causes increased ERK phosphorylation in quiescent vascular smooth muscle cells in a dose-dependent manner. Cells were exposed to 50, 100, 150 and in the case of SSc 5 200 µg/mL IgG. (TIF) [file pone.0100035.s001.tif]

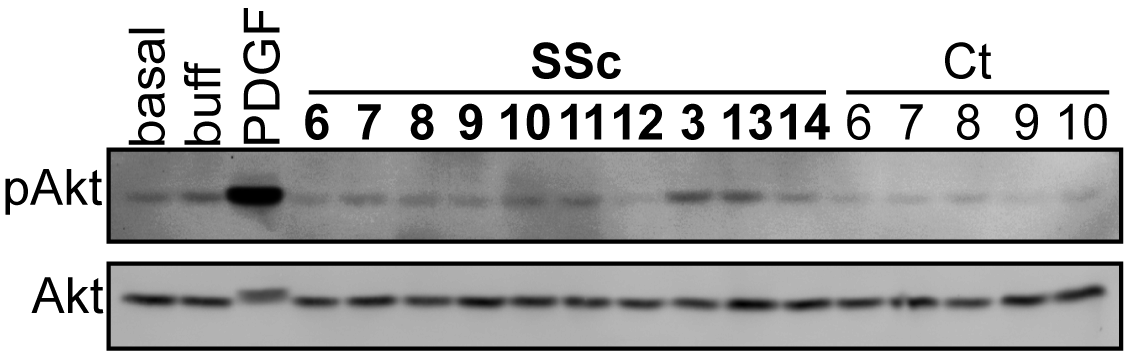

Supplement: Figure S2 — Effects of purified scleroderma (SSc) and control (Ct) IgG on signaling activity in a primary human fibroblast cell line (MRC5), after 5 minutes of exposure to 200 µg/mL purified IgG, 50 ng/mL PDGF, or the IgG buffer. (TIF) [file pone.0100035.s002.tif]

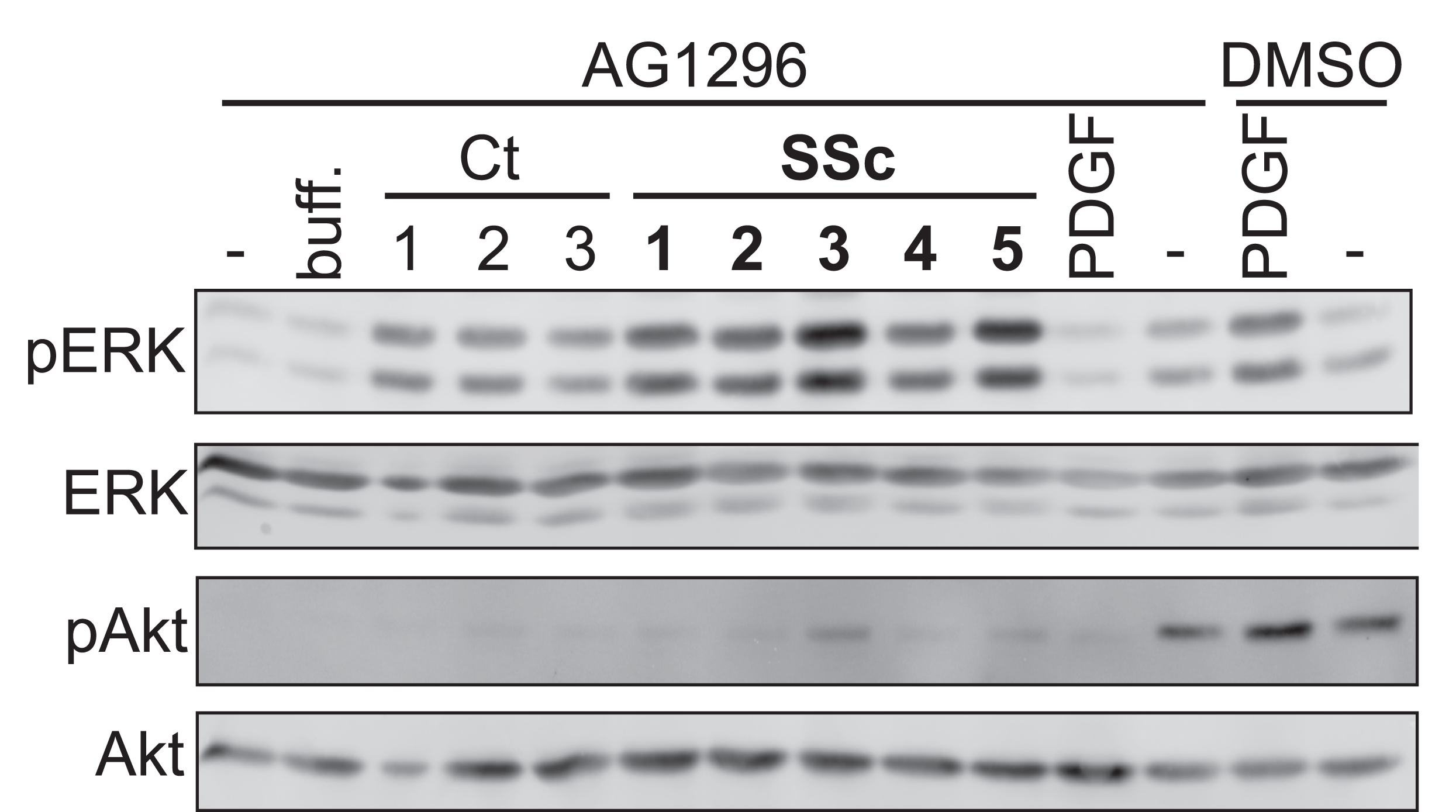

Supplement: Figure S3 — The platelet-derived growth factor receptor (PDGFR) inhibitor AG1296 does not inhibit the phosphorylation of ERK or Akt in vascular smooth muscle cells (VSMCs) stimulated with IgG from systemic sclerosis (SSc) patients. The inhibitor was effective in completely reducing the signal in response to stimulation with PDGF (compare PDGF/DMSO (lane 13) vs PDGF/AG1296 (lane 11)). Quiescent VSMCs were pre-treated for 30 minutes with vehicle (0.01% DMSO) or PDGFR-inhibitor AG1296 (5 µM) before stimulation with 50 ng/mL PDGF (P) or 200 µg/mL control (Ct) or scleroderma (SSc) IgG. (TIF) [file pone.0100035.s003.tif]

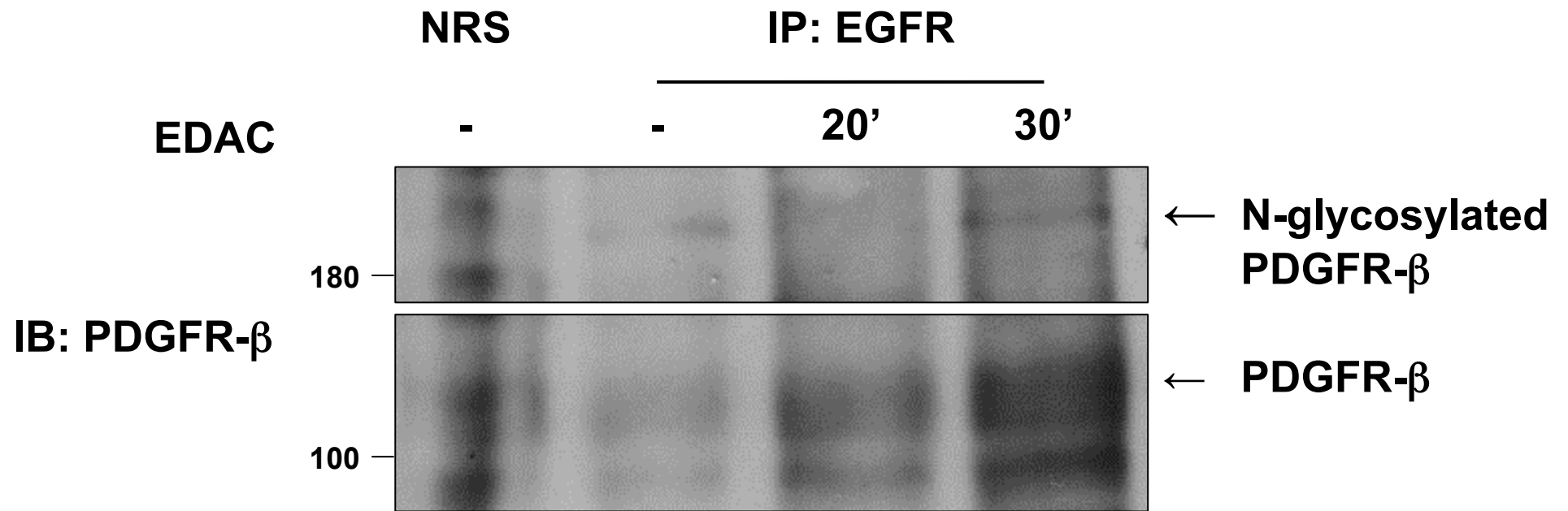

Supplement: Figure S4 — Constitutive heterodimerization of epidermal growth factor receptor (EGFR) and platelet-derived growth factor receptor (PDGFR) in quiescent vascular smooth muscle cells (VSMCs). Quiescent VSMCs were treated with the cross-linker 1-Ethyl-3-[3-dimethylaminopropyl]carbodiimide hydrochloride (EDAC) for 20 or 30 minutes. Cell lysates were prepared as described (Mol Cell Biol. 2001;21(19):6387-94C) and proteins were immunoprecipitated with anti-EGFR antibodies and immunoblotted for PDGFR-β. (PDF) [file pone.0100035.s004.pdf]
