# Supplementary material for: Systemic Sclerosis Immunoglobulin Induces Growth and a Pro-Fibrotic State in Vascular Smooth Muscle Cells through the Epidermal Growth Factor Receptor
Source: PLoS One. 2014 Jun 13;9(6):e100035. doi: 10.1371/journal.pone.0100035 (PMC4057313; doi:10.1371/journal.pone.0100035)
Supplement: Table S1 — Details of individual patient samples. ANA, type of anti-nuclear antibodies detected; PH, pulmonary hypertension; DSS, Medsger disease severity score (0 = normal, 1 = mild, 2 = moderate, 3 = severe, 4 = endstage); mRSS, modified Rodnan skin score; 1T, anti-topoisomerase I; 2R, anti-RNA Polymerase III; 30, none detected; 4n.a., data not available; 5C, anti-centromere; 6recent = having been stopped less than one year prior to blood draw; 7“not recent” = having been used at any time more than a year prior to blood draw; CS, corticosteroids; HCQ, hydroxychloroquine; MTX, methotrexate; 8stimulation index is calculated as (S-C)/(P-C)×100 where S, C, and P represent the normalized densitometric pERK1/2 band intensities of a given Sample, the negative Control and the Positive control, respectively. (PDF) [file pone.0100035.s005.pdf]

Sample	Sex	Age	ANA	PH	Active vascular ulcers	Peripheral vascular DSS	mRSS	Past use of immuno-suppressives	Stimu-lation Index[8]
1	F	24	T[1]	n.a.	+	3	22	CS: recent[6]	71
2	F	69	R[2]	+	+	4	43	None	79
3	F	45	R	-	+	2	29	MTX, HCQ: not recent[7]	69
4	F	43	T	-	-	3	6	None	35
5	F	60	R	-	-	1	18	None	69
6	F	46	T	-	+	3	4	None	0
7	F	.	0[3]	-	-	0	11	None	22
8	F	59	n.a.[4]	-	-	0	29	None	27
9	F	69	C[5]	-	-	0	17	None	49
10	F	25	n.a.	-	-	3	30	None	47
11	F	52	C	-	-	0	2	None	0
12	F	50	R	-	-	3	4	None	52

13	F	60	R	-	-	1	18	CS: not recent	82
14	F	75	C	-	-	1	14	None	15
15	F	32	T	-	+	n.a.	n.a.	None	16
16	F	38	0	-	+	n.a.	n.a.	None	29
17	F	33	R	-	-	n.a.	n.a.	None	10
18	F	30	T	-	-	n.a.	n.a.	None	100
19	F	65	C	-	-	2	13	None	186
20	M	53	T	-	+	3	14	MTX: recent	157
21	M	39	R	-	+	3	n.a.	None	58
22	F	60	R	-	n.a.	n.a.	n.a.	None	101
23	F	42	R	n.a.	-	2	30	None	17
